# Kimura's disease with eosinophilic panniculitis - treated with cyclosporine: a case report

**DOI:** 10.1186/1710-1492-6-5

**Published:** 2010-03-17

**Authors:** Davood Maleki, Alireza Sayyah, Mohammad Hossein Rahimi-Rad, Nasrin Gholami

**Affiliations:** 1 Department of Hematology, Urmia University of Medical Sciences, Imam Khomeini Hospital, Ershad street, Urmia, Iran; 2 Department of Internal Medicine, Urmia University of Medical Sciences, Imam Khomeini Hospital, Ershad street, Urmia, Iran; 3 Department of Pulmonology, Urmia University of Medical Sciences, Imam Khomeini Hospital, Ershad street, Urmia, Iran

## Abstract

Kimura's disease is a rare, benign, slow growing chronic inflammatory swelling with a predilection for the head and neck region and almost always with peripheral blood eosinophilia and elevated serum IgE levels. Here, we report a 25-year-old male patient with asthma, Reynaud phenomenon, eosinophilic panniculitis, bilateral inguinal lymphadenopathy and peripheral blood eosinophilia.

He responded initially to oral prednisolone with the subsidence of peripheral blood eosinophilia, asthma and the Reynaud phenomenon. But with tapering of prednisolone symptoms reappeared and hereby he was treated with cyclosporine. He has been symptom free for 6 months of follow up while taking cyclosporine 25 mg orally per day. Eosinophilia has resolved. This case shows that in addition to previously reported associations, Kimura disease may be associated with eosinophilic panniculitis and that cyclosporine could be effective in its treatment.

## Background

Kimura's disease (KD) is a rare, chronic inflammatory disorder of unknown cause. The most common clinical feature of this disease is an asymptomatic unilateral soft-tissue mass in the head and neck. Major salivary glands and lymph nodes may also be involved. Bilateral involvement is rare. Patients almost always have marked peripheral eosinophilia, and elevated serum IgE levels. Here, we report a 25-year-old male patient with asthma, Reynaud phenomenon, eosinophilic panniculitis, bilateral inguinal lymphadenopathy and peripheral blood eosinophilia.

## Case Presentation

A 25-year-old man presented with bilateral inguinal lymphadenopathy. He was suffering from frequent attacks of asthma for 2 years, and Reynaud phenomenon for a few months.

**Figure 1:**
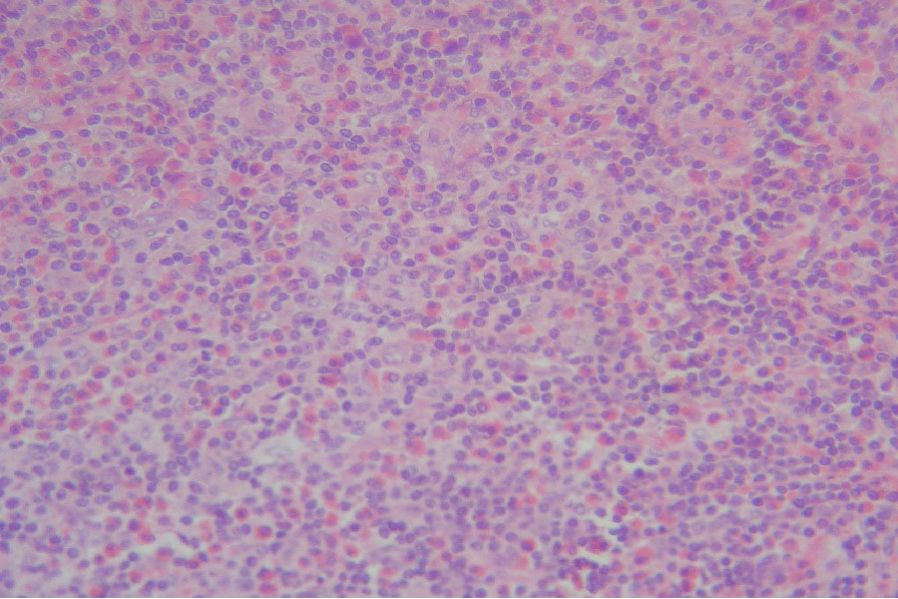
**Inguinal lymph node biopsy**. Note the diffuse interfollicular eosinophilic microabscesses in the lymph node.

Laboratory findings included a leukocyte count of 13850/mm^3 ^(Neutrophils: 4890 Lymphocytes: 1940 Eosinophils: 5980 (43%)), Hemoglobin = 16.7 g/dL and Platelet = 202000/mm^3^. C-reactive protein was negative and erythrocyte sedimentation ratio was 1 mm/h. Serum lactate dehydrogenase level was 322 IU/L. Liver function tests were normal. An excisional biopsy of inguinal lymph node showed lymphoid follicles with reactive germinal centers and well defined mantle zones, diffuse interfollicular eosinophilic microabscesses and apparent postcapillary venules, features associated with the diagnosis of KD (figure 1). He was discharged on treatment with oral prednisolone 1 mg/kg/day. One month later a follow up Complete blood count (CBC) showed evident decrease in eosinophilia: Leukocyte: 12480/mm^3 ^(Neutrophil: 10750 Lymphocyte: 1390 Eosinophil: 340 (about 4%)) and the Reynaud phenomenon was improved. Prednisolone was tapered and patient followed. Two months after tapering of prednisolone, he returned with 40% peripheral eosinophilia. He was prescribed with cyclosporine 200 mg twice daily orally.

After starting cyclosporine, he complained of headache and vertigo and rise in blood pressure. Renal function tests were normal and we decreased the dose of cyclosporine to 50 mg twice daily. In a follow up visit three months after the administration of cyclosporine, eosinophilia was less than 1% and the patient's asthma was in control without use of bronchodilators.

**Figure 2:**
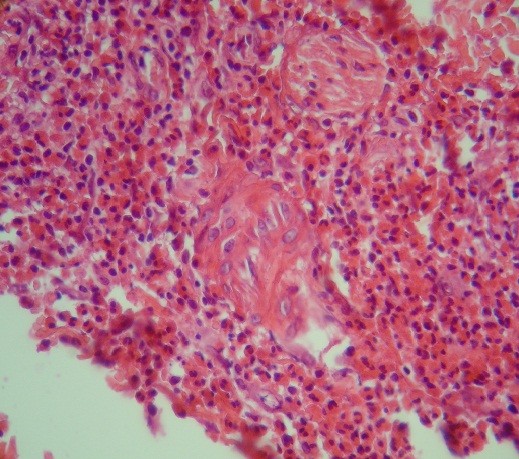
**Eosinophilic panniculitis**. Dense infiltration of lymphocytes, neutrophils and eosinophils (predominantly eosinophils) in perivascular and periadnexal areas.

In the next visit cyclosporine was discontinued and 15 days later he developed pruritic purple-red nodular lesions on his shins. Biopsy was taken from these lesions and cyclosporine 50 mg twice daily restarted. The result of skin biopsy was eosinophilic panniculitis with dense infiltration of lymphocytes, neutrophils and eosinophils (predominantly eosinophils) in perivascular and periadnexal areas, as well as involvement of subcutis septa and nodules of fatty tissue and aggregates of eosinophils between dermal collagen fibers (figure 2).

He is on a maintenance dose of 25 mg/day cyclosporine while there has been no more complaint of asthma attack, Reynaud phenomenon and skin lesions during the past 6 months.

## Discussion

Kimura's disease was first described in 1937 by Kim and Szeto in the Chinese literature and was later characterized by Kimura et al in 1948. This is a rare benign chronic inflammatory condition presenting with painless, slowly enlarging soft tissue mass, associated lymphadenopathy and peripheral blood eosinophilia. Soft tissue and lymph nodes of head and neck are involved typically. Bilateral involvement is rare. It mimics a number of lymphoproliferative and neoplastic conditions in its presentation [1].

KD usually occurs in the younger middle aged males between the second and third decades of life and is most common in Asian men. The onset of KD is insidious, following an indolent course, gradually increasing in size over months or years. The overall prognosis is good. Although spontaneous involution is rare, malignant transformation has not been documented [1].

The etiology and the pathogenesis of KD are unknown. The disease is classified as a benign reactive process. Allergic reactions, infections, and autoimmune reactions with an aberrant immune response have been suggested [2]. Complications such as atopic dermatitis, allergic rhinitis, asthma, and urticaria occur among patients.

The most common histologic features of KD include preserved nodal architecture; follicular hyperplasia with reactive germinal centers; well-formed mantle zones; eosinophilic infiltrates involving the interfollicular areas, sinusoidal areas, perinodal soft tissue, and subcutaneous tissue; and proliferation of postcapillary venules. The following can also be observed: proteinaceous deposits in germinal centers, vascularization of germinal centers, necrosis of germinal centers, polykaryocytes (Warthin-Finkeldey-type giant cells), eosinophils in germinal centers, eosinophilic folliculolysis, eosinophilic microabscesses, postcapillary venule proliferation, stromal sclerosis, perivenular sclerosis, or small eosinophilic granulomas. Sclerosis of variable degrees is often observed. Immunohistochemical staining with IgE shows a characteristic reticular staining pattern of germinal centers [3].

The anatomic site of lymph node involvement can be posterior auricular, cervical, inguinal (present patient), and epitrochlear lymph nodes, salivary gland involvement has also been reported [3]. KD can also present with middle mediastinal mass [4].

Patients almost always have marked peripheral eosinophilia, and elevated serum IgE levels [1]. The latter was not measured for our patient, but eosinophilia was prominent.

KD can be associated with mesangial proliferative glomerulonephritis, minimal change disease and membrane nephropathy [5,6]. Our patient had normal urine analysis and serum creatinine. Associations between KD and asthma, rash, Reynaud phenomenon [7], ulcerative colitis [8], temporal arteritis [6], eosinophilic myocarditis, lichen amyloidosus [9] and erytroderma [10], have been reported. Our patient was affected by two of these conditions, asthma and the Reynaud phenomenon. He also had eosinophilic panniculitis which has not been reported before.

The optimal treatment for KD is not well established. However, treatment should aim to preserve cosmetics and function while preventing recurrences and long-term sequels [11]. Therapeutic options have included surgery, radiotherapy, laser fulguration and steroids. There is a tendency for lesions to recur when steroid therapy is stopped. For recurrent lesions not responding to either modality, local irradiation (25-30 cGy) has been found effective [6]. Recent case reports have reported cyclosporine [6], azathioprine [12], pentoxifylline [11] and Imatinib [6] to be effective in the treatment of KD. We treated our patient initially with prednisolone, while cyclosporine showed effective to maintain recovery after steroid tapering. Cyclosporine inhibits calcium-dependent signaling pathways in transcription of IL-2 gene by affecting intracellular binding proteins. Decreased production of IL-2 inhibits T-cell proliferation and suppresses the immune response. Cytokines such as IL-4, IL-5 and IL-13 are said to have a role in production of IgE by B cells that is needed for differentiation of Th2 lymphocytes [13] and cyclosporine is reported to decrease the level of these cytokines. Senel et al [14] reported a combination of cyclosporine, azathioprine and prednisolone to be successful in treatment of KD and decreasing serum levels of soluble IL-2 receptor (sIL-2R), IL-4 and IL-5 consequently. Sato et al [13] also tried cyclosporine successfully in combination with prednisolone for treatment of an 11-year-old boy with relapsing KD after steroid tapering. They also showed a decrease in serum levels of sIL-2R, IL-4, IL-5, IgE and eosinophil count. In comparison to our case, these authors have experienced combination therapies including cyclosporine, but we treated our patient with cyclosporine alone after the relapse following prednisolone tapering. Katagiri et al [15] showed suppression of activity of KD by cyclosporine after incomplete surgical resection of the tumor and radiation therapy. High levels of IL-4, IL-5 and IL-13 mRNAs, peripheral blood eosinophil count and serum IgE level decreased after surgery and radiation and continued to decrease during cyclosporine therapy. It is suggested that Th2 lymphocytes and related cytokines are important in induction of eosinophilia and KD while cyclosporine acts against this condition by inhibiting Th2 cells and the cytokines [13].

Most of the case reports accepted local surgical excision as treatment of choice to preserve cosmetics and to relief of other symptoms. The exception is KD associated with nephritic syndrome, for which systemic treatment such as oral steroids is recommended. In the present case, lymphadenopathies were bilateral, of which only one on the left side was resected for diagnosis, and complete resection seemed impossible. On the other hand, he had Reynaud phenomenon and asthma as signs of a systemic disorder, making systemic therapy reasonable.

## Conclusions

There are some points of importance in this present case. Primarily it should be taken to consideration that unresponsive or steroid dependent asthma may be initial manifestation of an underlying condition such as KD. There are a few case studies showing cyclosporine to be useful in treatment of KD unresponsive to corticosteroids. We also observed dramatic response to cyclosporine in our patient who was dependent to high doses of steroid and kept our patient off steroids. We also reported eosinophilic panniculitis as a process in the course of KD that has not been reported previously. Although panniculitis occurred within a short time after discontinuation of cyclosporine and disappeared after reinstitution of cyclosporine, there is no certainty whether this condition is a manifestation of KD or just a coincidence. We hope this information to be useful in diagnosis and management of KD in other cases.

## Consent

Written informed consent was obtained from the patient for publication of this case report and any accompanying images. A copy of the written consent is available for review by the Editor-in-Chief of this journal.

## Competing interests

The authors declare that they have no competing interests.

## Authors' contributions

DM diagnosed the case and planned the treatment and medical follow ups. AS organized and finalized the manuscript, also prepared pathology pictures. MR did the pulmonary follow ups and contributed to the conclusion part. NG gathered the patient's history and drafted the manuscript. All authors read and approved the final manuscript.
